# Experimental Infection of Horses with West Nile virus

**DOI:** 10.3201/eid0804.010239

**Published:** 2002-04

**Authors:** Michel L. Bunning, Richard A. Bowen, Bruce C. Cropp, Kevin G. Sullivan, Brent S. Davis, Nieholas Komar, Marvin Godsey, Dale Baker, Danielle L. Hettler, Derek A. Holmes, Brad J. Biggerstaff, Carl J. Mitchell

**Affiliations:** * Centers For Disease Control and Prevention, Atlanta, Georgia; † Centers For Disease Control and Prevention, Fort Collins, Colorado, USA; ‡ United States Air Force; § Colorado State University, Fort Collins, Colorado, USA

**Keywords:** West Nile virus, flavivirus, arbovirus, horses, equine, New York, Aedes albopictus

## Abstract

A total of 12 horses of different breeds and ages were infected with West Nile virus (WNV) via the bites of infected Aedes albopictus mosquitoes. Half the horses were infected with a viral isolate from the brain of a horse (BC787), and half were infected with an isolate from crow brain (NY99-6625); both were NY99 isolates. Postinfection, uninfected female Ae. albopictus fed on eight of the infected horses. In the first trial, Nt antibody titers reached >1:320, 1:20, 1:160, and 1:80 for horses 1 to 4, respectively. In the second trial, the seven horses with subclinical infections developed Nt antibody titers >1:10 between days 7 and 11 post infection. The highest viremia level in horses fed upon by the recipient mosquitoes was approximately 460 Vero cell PFU/mL. All mosquitoes that fed upon viremic horses were negative for the virus. Horses infected with the NY99 strain of WNV develop low viremia levels of short duration; therefore, infected horses are unlikely to serve as important amplifying hosts for WNV in nature.

## Introduction

West Nile virus (WNV), a flavivirus related to Japanese encephalitis, St. Louis encephalitis, and Murray Valley encephalitis viruses, was responsible for outbreaks of encephalomyelitis in humans and horses in New York (NY) during 1999 (1). The recognition of WNV in North America during 1999 was the first indication this virus was present in the Western Hemisphere. Continuing widespread virus activity in the northeastern USA during 2000 (2) suggests that WNV has become endemic. Previously, this virus was known from Africa, Europe, and south Asia; a subtype, Kunjin, is recognized in Australasia. During the peak transmission season, WNV cycles mainly between mosquito and wild bird species that differ according to geographic area. Before 1999, WNV had been reported to cause equine encephalomyelitis in Egypt (3), the Rhone River delta of France (4), Morocco (5), Israel (6), and Italy (7). Most equine infections were thought to result in mild clinical disease or inapparent infections, with only occasional cases of severe disease. However, during the 1999 epizootic, high illness and death rates in a cluster of equine cases centered around Riverhead, Long Island, Suffolk County, NY, indicated that not all infections resulted in mild disease (8). Within a radius of 10 km, 36 (43%) of 83 horses sampled from the Riverhead area were seropositive for WNV, and the clinical attack rate among the seropositive animals was 42%. The death rate for the 22 infected horses from Suffolk County was 36%. These observations raised the question of whether horses might be serving as amplifying hosts for WNV, exacerbating public health and veterinary problems.

Previous WNV experimental infection studies in equids are few, and attempts to induce and study clinical disease caused by WNV in equids have yielded equivocal results (3,9). In studies by Joubert et al. (10) and Oudar et al. (11), fever developed in four of nine equids (one jenny, one horse, and seven foals) after simultaneous needle inoculation of WNV by the subcutaneous and intravenous routes. In three of the four foals that became febrile, frank meningoencephalomyelitis and specific histopathologic lesions developed in central nervous system (CNS) tissue (12). One of 12 horses in our study developed encephalomyelitis (13).

Previous studies did not resolve the question of whether WNV-infected equids produce viremia levels of sufficient magnitude and duration to infect vector mosquitoes. Schmidt and Mansoury (3) reported transient (1-day) trace amounts of WNV in the blood of two of six donkeys infected by needle inoculation, but detectable levels of viremia did not develop in three horses in the study. Viremia titers and duration were not reported quantitatively in the studies by Joubert et al. (10) and Oudar et al. (11). A study conducted by U.S. Department of Agriculture (USDA) staff (J.Lubroth, pers. comm.) also reported low levels of viremia (< 102.5 TCID50 per mL) in four horses infected by needle inoculation of a strain of WNV isolated from a horse during the 1999 epizootic.

Because of the lack of definitive studies on the potential for equines to serve as amplifying hosts for WNV following vector-borne transmission and the implications for public health and veterinary concerns, studies were needed of horses infected by mosquito bite with virus strains isolated during the 1999 outbreak in New York. Accordingly, we investigated the course of clinical disease (13), viremia levels and antibody responses, and the potential of viremic horses to infect vector mosquitoes.

## Methods

### Equine selection and examinations

Mares and geldings of varying ages and breads were used (Table 1). The horses were screened and shown to be negative for neutralizing antibodies to WNV and St. Louis encephalitis viruses (SLEV). Two days before infection, they were moved into a biocontainment building at Colorado State University and maintained under biosafety level-3 conditions for the duration of the project. The horses were fed mixed grain and hay twice a day. The horses were euthanized by pentobarbital overdose at varying times after infection (Table 1), necropsies were performed, and the carcasses were incinerated in the containment facility.

**Table 1:** Virus strain, age, sex, and day animal was euthanized after inoculation, May–July 2000

**Identification no.**	**Virus strain**	**Age(yr)**	**Sex**	**Euthanized (days after inoculation)**
**1**	**BC787**	**14**	**Male**	**13**
**2**	**BC787**	**11**	**Female**	**12**
**3**	**NY99-6625**	**7**	**Female**	**12**
**4**	**NY99-6625**	**11**	**Female**	**13**
**9**	**BC787**	**11**	**Female**	**>90**
**10**	**BC787**	**10**	**Female**	**>90**
**11**	**BC787**	**13**	**Male**	**9**
**12**	**BC787**	**4**	**Male**	**>90**
**13**	**NY99-6625**	**5**	**Female**	**>90**
**14**	**NY99-6625**	**4**	**Female**	**>90**
**15**	**NY99-6625**	**6**	**Male**	**>90**
**16**	**NY99-6625**	**18**	**Female**	**27**

The horses were examined for signs of disease twice a day. Their body temperatures were recorded twice a day from day 0 (day of infection) to day 10, then daily through day 28, unless they were euthanized earlier. Pulse and respiration rates were recorded daily for the first 2 weeks after infection. Blood was collected for serum twice a day from day 0 through day 14, then daily through day 28, and twice a week for the duration of the project. Oral and rectal swabs were obtained daily from days 0 through 10, and the samples were placed into 1 mL of BA-1 medium (M-199 salts, 1% bovine serum albumin, 350 μ/L sodium bicarbonate, 100 units/mL penicillin, 100 μ/L streptomycin, and 1 μ/L amphotericin in 0.05 M Tris, pH 7.6) and stored frozen for virus isolation. Blood samples were analyzed daily from day 0 through day 10 by using a QBC-V hematology analyzer (Clay Adams, Becton, Dicksinson and Company, Franklin Lakes, NJ). The methods used for histopathology and immunocytochemistry have been described (R. Bowen, submitted for pub.).

Horses 5 through 9 were used to test a vaccine for WNV and were not part of this research project.

### Mosquito Infection and Feeding Trials

Mosquitoes used for transmitting WNV to susceptible horses by bite were infected with either of two strains of WNV isolated from New York animals infected during the 1999 outbreak. Strain BC787 (1 passage in suckling mouse brain) was isolated from the brain of a horse, and NY99-6625 (1 passage in Vero cell culture) was isolated from the brain of a crow.

The Aedes albopictus mosquitoes used in these experiments were from a colony strain from Lake Charles, LA. Mosquitoes were reared in an insectary maintained at 26.7°C (± 0.5°) in approximately 80% relative humidity and with a long photophase (light:dark 16:8). Larvae were fed liver powder and rabbit chow as desired. Cohorts of 3- to 5-day-old adult female Ae. albopictus were inoculated intracoelomically with either of the two virus strains, placed in separate cages, given 5% sucrose for maintenance, and incubated under the insectary conditions described above. The virus dose per mosquito, based on appropriate dilutions of stock virus of known titer, was estimated to be approximately 170 Vero PFUs. Sucrose was withheld prior to feeding on equines.

Infected mosquitoes were allowed to feed on horses in two separate trials. A pilot experiment was conducted with four horses to determine probable periods of peak viremia before a more comprehensive experiment was conducted, which involved the feeding of large numbers of uninfected (recipient) mosquitoes. On day 8 postinoculation (May 10, 2000), caged infected mosquitoes were placed in a securely taped styrofoam container and transported to the equine holding facility. Feeding was accomplished by holding a cage of mosquitoes, with a gloved hand, against a shaved area immediately behind the left shoulder of each horse. Based on viremia profiles from the initial trial, a second trial was begun on July 10, 2000, in which infected mosquitoes that had been incubated 12 days postinoculation were allowed to feed on eight horses. Each horse was exposed to the mosquitoes for 5 minutes in each trial.

After feeding, the caged mosquitoes were sealed in a styrofoam container that was rinsed externally with diluted bleach and then transported to a laboratory. Mosquitoes were anesthetized with CO2 and sorted on wet ice. Most blood-fed specimens were placed in stoppered vials and frozen at -70°C until they were triturated and tested for virus. Mosquitoes were ground individually in 1 mL each of BA-1 diluent in TenBroeck (Wheaton Science Products, Millville, NJ) tissue grinders. Samples were centrifuged for 4 minutes at 20,000 x g in a refrigerated microcentrifuge. Supernatants were poured into screw-cap vials, which were kept on wet ice until serial dilutions were made, and the samples were tested for virus by plaque assay.

During the second experiment, on days 3, 4, and 5 after infected mosquitoes had fed on the horses, uninfected 3- to 7-day-old Ae. albopictus females were permitted to feed on the 8 horses in lots of approximately 40 mosquitoes per cage. In addition, 3 lots of recipient mosquitoes were fed on horse 11 on days 8 and 9 postinfection, after the horse showed signs of clinical illness. Recipient mosquitoes were allowed to feed in an area behind the right shoulder of the animals on the side opposite from where the infected mosquitoes had fed. Fed mosquitoes were transported to a laboratory and sorted with a mechanical aspirator, and each cohort was placed in a separate 0.2-L cage. Blood-engorged mosquitoes were given 5% sucrose for maintenance, and the 0.2-L cages were placed inside larger cages and incubated as described. Recipient mosquitoes that fed on horses on days 3 and 4 postinfection were incubated for 10 days. The 8 cohorts of recipient mosquitoes fed on day 5 postinfection were incubated only 7 days because a large number had died. Following incubation, mosquitoes were immobilized by being chilled briefly and were placed in tubes while still alive. They were then frozen at -70°C until processed and tested for virus by plaque assay. All mosquitoes fed on days 3 to 5 postinfection were individually disrupted by sonic energy (13) in 1 mL of BA-1 diluent and centrifuged at 4°C for 15 min at 1,500 x g. The supernatants were frozen at -70°C until tested by plaque assay. The three lots of mosquitoes that fed on the sick horse on days 8 and 9 postinfection were processed and tested in three pools, titurated in 1.8 mL of BA-1, and centrifuged for 4 minutes at 20,000 x g in a refrigerated microcentrifuge.

### Mosquito Saliva Collection

Some of the WNV-infected mosquitoes used for horse challenge were chilled at 4°C for 5 minutes and held on ice. Mosquitoes were immobilized by removing their legs and wings. Individual mosquito proboscises were placed in a capillary tube containing Type “B” immersion oil. Saliva was collected in the oil-filled tube (14). Capillary tubes containing individual mosquito saliva were placed in 1.7-mL centrifuge tubes containing 200 μL of BA-1 diluent and centrifuged (105 rpm for 2 min). Serial dilutions were made, and the contents were inoculated into Vero cell culture six-well plates. A double agarose overlay was used, with the second overlay containing neutral red, and plaques were counted on days 4 and 5 postinoculation.

### West Nile Equine IgM Capture ELISA

The WNV equine immunoglobulin (Ig)M capture enzyme-linked immunosorbent assay (ELISA) is a microtiter plate format assay designed to detect IgM antibodies to WNV. Unused 96-well plates (Immunolon II; Dynatech Laboratories, Chantilly, VA) were coated overnight at 4°C with 75 μL of goat anti-horse IgM antibody (Kirkegaard & Perry Laboratories, Gaithersburg, MD) in carbonate buffer (0.015 M NaCO3, 0.035 M NaHCO3, pH 9.6) at a 1:1,000 dilution. For quality control, 36 outside wells of the plate were not used in the test. After the coating buffer was decanted, 200 μL of blocking buffer (phosphated-buffered saline [PBS], 0.05% Tween 20, 5% dry milk) was added to each well, and the wells were incubated at room temperature for at least 30 minutes. After the mixture was washed five times with wash buffer (PBS, 0.05% Tween 20), 50-μL samples of horse serum, diluted 1:400 in wash buffer, were applied; each sample was added to 6 wells. Positive and negative control equine sera were included on each plate, yielding a final capacity of eight test samples per microtiter plate. Diluted serum was incubated for 1 hour at 42°C. After the serum was washed five times, 50 μL of WNV tissue culture antigen (CDC Cat. No. VA2395), diluted 1:300 in wash buffer, was added to half the wells to which diluted serum was added. And 50 μL normal control tissue culture antigen (CDC Cat. No. VB2396), diluted 1:300 in wash buffer, was added to the remaining wells so that each test serum sample had three viral antigen wells and three normal antigen wells. The antigen was incubated overnight at 4°C. After the samples were washed five times, 50 μL of horseradish peroxidase, conjugated with a monoclonal antibody to WNV (6B6C-1 CDC; ABD [Arboviral Branch] reference reagent) and diluted 1:2000 in blocking buffer, was added to each well and incubated for 1 hour at 42°C. After the wells’ contents were washed 10 times with wash buffer, 75 μL tetramethylbencidine (TMB) substrate (GibcoBRL, Life Technologies Inc, Gaithersburg, MD) was added to each well. After incubation with the substrate for exactly 10 minutes, the reaction was stopped by adding 50 μL 1N H2SO4. The OD of each well was measured at 450 nm, and results were calculated as follows:

The Final A450nm = [A450 nm of test sera on West Nile antigen] – [A450nm of normal sera on West Nile antigen]

### Neutralization Assay

Serum samples were tested for virus-neutralizing (Nt) antibodies to WNV (NY99-4132 strain, 1 Vero passage) by the plaque-reduction neutralization test in Vero cell culture. Earlier serum samples were also tested for Nt antibodies to SLEV (TBH-28 strain, passage history unknown). Briefly, diluted serum was heat inactivated at 56°C for 30 minutes and mixed with an equal volume of a virus preparation BA-1 containing 8% normal human serum, so that the number of infectious virus particles in the final dilution was approximately 100/0.1 mL. A volume of 0.1 mL was then injected onto a Vero cell monolayer and processed as for the plaque assay. Samples were screened by testing once at a final dilution (after mixture with challenge viruses) of 1:10. Any sample that neutralized the challenge virus dose at a level of >70% was confirmed by testing in duplicate and titrated by serial twofold dilutions. Neutralization at a level of >90% was considered positive for each dilution. Horses having preexposure sera that neutralized SLEV at >70% were excluded from the study. Sera from horses 1 to 4 were challenged with approximately 300 PFU of WNV (Horse Brain WN Isolate # BC 787), and sera from horses 9 to 16 had an average of 86 PFU of WNV added.

### Plaque Assay

Virus concentration in serum samples, mosquito homogenates, and tissue homogenates was measured by titration in a plaque assay. Briefly, 0.1 mL of sample was added to a monolayer of Vero cells in a six-well cell culture plate (Costar, Corning Incorporated Life Science, Acton, MA) and incubated 1 hour at 37°C in 5% CO2. Cells were overlaid with 3 mL per well of 0.5% agarose in M-199 medium, supplemented with 350 mg/L of sodium bicarbonate, 29.2 mg/L of L-glutamine, and antibiotics. After 48 hours of additional incubation, a second 3-mL 0.5% agarose overlay, containing 0.004% neutral red dye, was added for plaque visualization. The plaques were scored on days 3, 4, and 5 of incubation.

## Results

In the first infection trial, 12 to 17 infected mosquitoes fed on each of four horses. Virus titrations were done on 10 blood-engorged mosquitoes from each cohort that fed. All of the 40 mosquitoes tested were infected. Virus titers ranged from 106.6 to 107.9 Vero cell PFU per mosquito and averaged 106.8, 107.2, 107.3, and 107.4 PFU per mosquito in the cohorts that fed on horses 1 to 4, respectively. Mosquitoes infected with the WNV strain isolated from a horse were fed on horses 1 and 2, and mosquitoes infected with a strain isolated from a crow were fed on horses 3 and 4 (Table 1). Serum samples (drawn twice a day from day 0 through days 12 or 13) were tested for virus. Horse 3 did not develop a detectable level of viremia, and horse 1 had viremia detectable only in the afternoon of day 3 post-infection titer of 101.3 PFU/mL. Horse 2 had peak titers of 101.3, 102.2 and 102.2 PFU/mL on days 3 to 5, respectively. Horse 4 had titers ranging from 101.0 PFU/mL in the afternoon of day 2 to 101.3 PFU/mL on day 4.

On the basis of these viremia profiles, we decided to feed uninfected mosquitoes on infected horses in the next trial on days 3 to 5 postinfection to determine whether viremia levels were sufficient to infect susceptible mosquitoes. None of these horses developed fever, hematologic abnormalities, or signs of clinical disease. Horses 1, 2, 3, and 4 were euthanized, and necropsies were performed on days 14 and 16 postinfection. Histopathologic lesions indicative of WNV infection were not observed. Virus was not isolated from any tissues from these animals, and viral antigens were not detected in brain, spinal cord, or other tissues by immunocytochemical tests.

In the second trial, 7 to 14 infected mosquitoes fed on each of the eight horses. Virus titrations were done on five mosquitoes from each cohort, and 100% of the mosquitoes tested were infected. Virus titers ranged from 106.5 to 108.0 Vero cell PFU per mosquito and averaged 10 7.5,107.3, 107.3, 107.3, 107.6,107.3, 107.7, and 107.4 PFU per mosquito in the cohorts that fed on horses 9 numbered to 16, respectively. Mosquito saliva was collected from 10 infected mosquitoes used in the second trial, and virus titers ranged from 101.3 to 102.5 PFU per sample of saliva.

Mosquitoes infected with a WNV strain isolated from a horse were fed on horses 9 numbered to 12 and mosquitoes infected with a strain isolated from a crow were fed on horses numbered 13 to 16 (Table 1). Seven of the eight horses developed detectable levels of viremia, and all virus-positive serum samples were obtained during days 1 to 6 (Table 2). Virus titers ranged from 101.0 PFU/mL of serum, the lowest level detectable in our assay, to 103.0 PFU/mL (in horse 13 in the p.m. of day 3).

**Table 2:** Postinfection levels of West Nile viremia in horses, May–July 2000a

	**Viremia levels (Log-10 Vero cell PFU/mL serum)**							
	**Horse**							
**Day**	**9**	**10**	**11**	**12**	**13**	**14**	**15**	**16**
**1 (a.m.)**	**1.3**	**-**	**-**	**-**	**-**	**-**	**-**	**-**
**1 (p.m.)**	**-**	**1.0**	**-**	**-**	**-**	**-**	**-**	**-**
**2 (a.m.)**	**-**	**1.3**	**-**	**-**	**-**	**-**	**-**	**-**
**2 (p.m.)**	**-**	**1.0**	**-**	**1.0**	**-**	**-**	**-**	**1.0**
**3 (a.m.)**	**2.1**	**1.5**	**1.0**	**-**	**1.0**	**-**	**2.2**	**-**
**3 (p.m.)**	**2.3**	**1.3**	**-**	**-**	**3.0**	**-**	**-**	**1.9**
**4 (a.m.)**	**2.4**	**1.6**	**2.5**	**1.5**	**1.3**	**-**	**1.3**	**2.1**
**4 (p.m.)**	**1.9**	**1.5**	**1.9**	**1.0**	**1.3**	**-**	**-**	**2.0**
**5 (a.m.)**	**1.6**	**1.5**	**2.7**	**1.0**	**1.3**	**-**	**-**	**2.5**
**5 (p.m.)**	**-**	**1.6**	**2.5**	**-**	**1.3**	**-**	**-**	**2.7**
**6 (a.m.)**	**-**	**1.6**	**2.1**	**-**	**-**	**-**	**-**	**2.3**
**6 (p.m.)**	**-**	**1.6**	**2.1**	**-**	**-**	**-**	**-**	**2.0**

**^a^Horses 9 to 12 were infected by bites of mosquitoes inoculated 12 days earlier with WN virus strain isolated from a horse; horses 13 to 16 similarly infected with strain isolated from a crow; dash indicates viremia level was too low to be detectable or was absent.**

Twenty-four lots of uninfected recipient mosquitoes were fed on seven infected horses in the mornings of days 3 to 5 postinfection. Virus titers in the seven viremic horses ranged from 101.0 PFU/mL to 102.7 PFU/mL during the times mosquitoes were fed. The feeding success among uninfected mosquitoes was 92%, 81%, and 71% on days 3 to 5, respectively. Following incubation, only live mosquitoes were saved for testing. Survival rates for the mosquitoes that fed on days 3, 4, and 5 were 256(87%) out of 293, 246(92%) out of 266, and 150(64%) out of 236, respectively. All 652 mosquitoes that fed during the 3 days and survived the incubation periods were tested individually for the presence of virus and found to be negative. In addition, after horse number 11 developed clinical symptoms compatible with encephalomyelitis three lots of mosquitoes were fed on animal number 11 on day 8 (a.m. and p.m.) and day 9 (a.m.). The mosquitoes were incubated for 10 days and tested for virus infection in three pools of 30, 41, and 44 specimens. All were negative for the presence of virus.

The only horse from the entire study to show clinical signs of disease was horse 11, which became febrile and showed neurologic signs beginning 8 days after infection. This mare progressed to severe clinical disease within 24 hours and was euthanized on day 9. She had a severe encephalomyelitis and relatively high titers of virus (104.0 to 106.8 PFU/tissue) in several areas of the brain and spinal cord. Horse 16 was euthanized on day 30 because of a preexisting respiratory condition unrelated to WNV infection. Horses 9, 10, 12, 13, 14, and 15 were monitored for 91 days after infection. None showed signs of disease at any time during that period, nor was WNV isolated from any of the serum samples collected biweekly through day 91. Oral and rectal swabs collected daily from days 0 through 10 were negative for virus.

All 12 horses became infected with WNV after being bitten by infected mosquitoes as evidenced by clinical encephalomyelitis in horse 11 and Nt antibody titers in the others. In the first trial, Nt antibody titers >1:10 were first detected in horses 1 to 4 on days 8 to 10 postinfection. Titers in the samples drawn on days 12 and 13 were >1:320, 1:20, 1:160 and 1:80 for horses 1 to 4, respectively. These horses were euthanized and underwent necropsy between days 14 and 16. In the second trial, the seven horses that remained well developed Nt antibody titers >1:10 between days 7 and 11 post infection. Peak titers in seven horses that did not exhibit clinical illness were reached between days 9 through 13. No samples were tested between days 13 and 27, by which time the titer of horse 16 was >1:1,280. Titers for horses 9, 10, and 12 to 15 on day 31 postinfection were 1:320, 1:160, 1:160, 1:160, 1:40, and 1:80, respectively.

**Figure 1:**
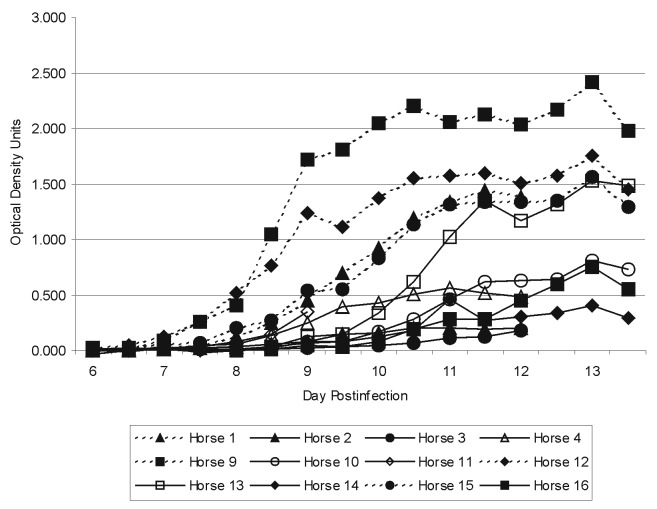
Absorbance Attributed to specific West Nile virus (WNV) Antigen immuglobulin (Ig) M Interaction in the West Nile Equine IgM Capture enzyme-linked immunosorbent assay (ELISA).

**Figure 2:**
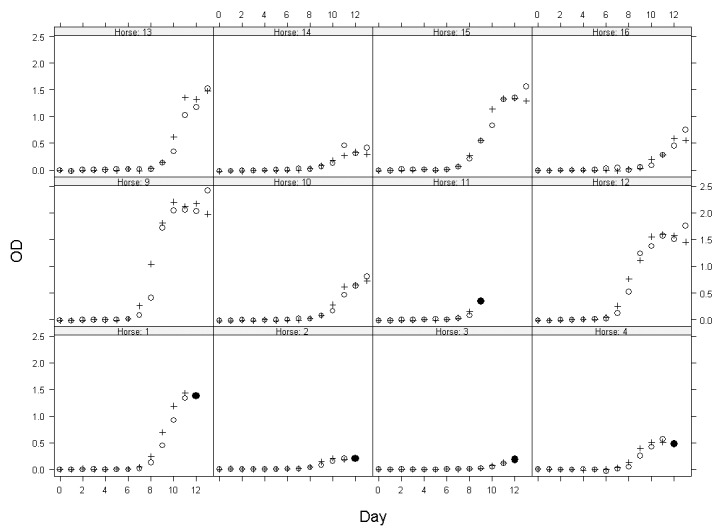
The open circle (o) is the a.m. reading, the plus (+) is the p.m. reading, and the filled-in circle is the last reading (all a.m., hence the circle) before the horse was euthanized. Each number corresponds to the horse number.

The IgM capture ELISA results are summarized in Figure 1 and Figure 2. Specific IgM levels in some horses began to rise perceptibly by day 7 postinfection and continued to increase up to day 13. Horses 3 and 14, which did not have detectable levels of viremia, showed weak responses. Horse 11 also did not have detectable levels of viremia and was euthanized on day 9.

## Discussion

Ae. albopictus mosquitoes were used in these experiments because this species was known to be susceptible to WNV infection by mouth and by intracoelomic inoculation and to be capable of transmitting the virus by bite (15–17). In addition, information was available on the replication of WNV in intracoelomically inoculated Ae. albopictus, incubated under conditions identical to ours, which showed that peak titers were reached by day 5 postinoculation (K. Gottfried, pers. comm.). WNV has been isolated recently from Ae. albopictus in New York (18).

The key question addressed in these studies was whether horses develop viremia of sufficient magnitude to serve as amplifying hosts of WNV. The low titers we observed are similar to those seen by Schmidt and Mansoury in Egypt (3). Joubert et al. (19) conducted studies in France; however, they did not report actual titers but rather categories of viremia (weak, moderate, or strong), based on intensity and duration. Recent unpublished work also is consistent with these findings (J. Lubroth, pers. comm.). Our finding of 1(8.3%) of 12 clinical cases among the experimentally infected horses agrees closely with the results reported for naturally acquired WNV infections among horses in Camargue, France, where the clinical attack rate among approximately 500 free-ranging equines was estimated to be 10% (10).

The highest viremia titer in horses fed on by the recipient mosquitoes was approximately 460 Vero cell PFU/mL in horse 11 during the morning of day 5 postinfection (Table 2). This mare was the only animal in our study to develop clinical encephalomyelitis. Although relatively high titers of WNV were found in several areas of the brain and spinal cord, titers in the horses' blood were not extraordinary. Based on an estimated bloodmeal volume of 5 μL/mosquito, the average virus dose ingested by mosquitoes feeding on this animal was 2.3 PFU. Twenty-two mosquitoes from the cohort that ingested this estimated dose survived to be tested, and all were WNV-negative. We did not feed mosquitoes during the evening of day 3 postinfection when horse 13 was circulating 103.0 PFU/mL, or the equivalent of 5 PFU/bloodmeal. Whether this dose, the highest viremia titer recorded in the horses, would have been sufficient to infect our strain of Ae. albopictus is unknown. Jupp (20) reported that 41% of a South African strain of Culex univittatus became infected after feeding on a viremic chick circulating 102.9 suckling-mouse intracerebral lethal-dose50 (SMICLD50)/mL of WNV. Further, estimates were made of the viremia levels necessary to infect 10% of vector mosquitoes. These 10% infection thresholds, expressed as titers found in viremic chicks, for local strains of Cx. univittatus, Cx. pipiens, Cx. quinquefasciatus, and Cx. theileri were <102.7, 102.7,102.7 and<104.1 adult-mouse LD50/mL, respectively. The results are somewhat difficult to interpret because not all species were fed on low-titered viremias, and because both adult and infant mice, which differ in their susceptibility to WNV infection, were used for some titrations. Jupp (21) also noted discrepancies in infection thresholds related to the manner of feeding, using blood-virus mixtures fed through membranes or via blood-soaked cotton, as opposed to feeding on viremic animals. Interestingly, these differences were apparent in Cx. pipiens and Cx. quinquefasciatus but not in the other two species. Results of earlier studies in which data were obtained by artificial feeding techniques should be viewed with caution, at least for the species mentioned. We are unaware of other information on the infection thresholds of species and strains of proven and potential vectors of WNV.

Akhter et al. (17) fed C. tritaeniorhynchus and Cx. quinquefasciatus on five viremic chicks that were circulating 104.9 to 105.3 SMICLD50 of WNV. All Cx. tritaeniorhynchus (n=100) became infected, and 59% to 90% of the Cx. quinquefasciatus (n=85) did so. Turell et al. (22) fed a variety of mosquito species from the eastern USA on viremic chicks circulating WNV at titers of approximately 105.2 Vero cell PFU/mL and 107.0(+ 0.3) PFU/mL. Based on an estimated bloodmeal volume of 5 μL/mosquito, individual mosquitoes, which fed on these chicks, would have ingested virus doses of about 800 and 40,000 to 160,000 PFU, respectively. Infection rates in mosquito species in these cohorts ranged from 0% to 17% and 0% to 92%. For other arboviruses, a dose-response relationship exists between the titer of the infective meal and the ability of vector mosquitoes to transmit (23). An Israeli strain of the molestus biotype of Cx. pipiens transmitted WNV to infant mice after feeding on high-titered blood/virus mixtures but not when the titer was 102.8/0.3 mL (104.3/mL) (24). Jupp (21) noted that a reduction in the infecting WNV titer from 106.5 to 104.3 SMICLD50 caused a decrease in the transmission rate from 89% to 33% in Cx. univittatus. Therefore, if the low-level viremias observed in our study were to prove sufficient to infect mosquito species shown to be more susceptible to by mouth infection than Ae. albopictus, any individuals that did become infected on such low doses of WNV would be less likely to transmit the virus.

The results of the equine IgM-capture ELISA for WNV antibody indicate that this is a simple and efficient method for detecting antibody at about the same time that neutralizing antibody can be detected (Figure 2). The results of the Nt tests are unremarkable and consistent with those from previous studies (3,19).

Our limited work, and that of others cited above, support the conclusion that horses infected with WNV develop viremias of low magnitude and short duration and that infected horses are unlikely to serve as important amplifying hosts for WNV in nature.
